# Heme oxygenase-1 regulates cell proliferation via carbon monoxide-mediated inhibition of T-type Ca^2+^ channels

**DOI:** 10.1007/s00424-014-1503-5

**Published:** 2014-04-18

**Authors:** Hayley Duckles, Hannah E. Boycott, Moza M. Al-Owais, Jacobo Elies, Emily Johnson, Mark L. Dallas, Karen E. Porter, Francesca Giuntini, John P. Boyle, Jason L. Scragg, Chris Peers

**Affiliations:** 1 Division of Cardiovascular and Diabetes Research, LIGHT, Faculty of Medicine and Health, University of Leeds, Clarendon Way, Leeds, LS2 9JT UK; 2 School of Pharmacy, University of Reading, Reading, RG6 6UB UK; 3 School of Pharmacy and Biomolecular Sciences, Liverpool John Moores University, Liverpool, UK

**Keywords:** Heme oxygenase, Carbon monoxide, Calcium channel, Proliferation, Vascular smooth muscle

## Abstract

Induction of the antioxidant enzyme heme oxygenase-1 (HO-1) affords cellular protection and suppresses proliferation of vascular smooth muscle cells (VSMCs) associated with a variety of pathological cardiovascular conditions including myocardial infarction and vascular injury. However, the underlying mechanisms are not fully understood. Over-expression of Ca_v_3.2 T-type Ca^2+^ channels in HEK293 cells raised basal [Ca^2+^]_i_ and increased proliferation as compared with non-transfected cells. Proliferation and [Ca^2+^]_i_ levels were reduced to levels seen in non-transfected cells either by induction of HO-1 or exposure of cells to the HO-1 product, carbon monoxide (CO) (applied as the CO releasing molecule, CORM-3). In the aortic VSMC line A7r5, proliferation was also inhibited by induction of HO-1 or by exposure of cells to CO, and patch-clamp recordings indicated that CO inhibited T-type (as well as L-type) Ca^2+^ currents in these cells. Finally, in human saphenous vein smooth muscle cells, proliferation was reduced by T-type channel inhibition or by HO-1 induction or CO exposure. The effects of T-type channel blockade and HO-1 induction were non-additive. Collectively, these data indicate that HO-1 regulates proliferation via CO-mediated inhibition of T-type Ca^2+^ channels. This signalling pathway provides a novel means by which proliferation of VSMCs (and other cells) may be regulated therapeutically.

## Introduction

Vascular smooth muscle cells (VSMCs) control vascular tone (and hence blood flow and distribution) through regulated contraction which is highly dependent on Ca^2+^ influx, primarily via voltage-dependent L-type Ca^2+^ channels [4, 21, 33, 48, 50, 54]. VSMCs are not terminally differentiated and can undergo adaptive phenotypic changes: their ability to become non-contractile, proliferative cells is an important factor in both developmental vasculogenesis and vascular repair [35, 36, 52]. However, this switch to a proliferative state is also important in pathological situations such as atherosclerosis, restenosis, neointimal hyperplasia and hypertension [19, 36]. Given the impact of such cardiovascular diseases on global health, a greater understanding of the mechanisms underlying this phenotypic change in VSMCs has tremendous potential to reveal novel therapeutic strategies.

Although T-type Ca^2+^ channels are expressed in VSMCs, their role in vasoconstriction is unclear (see [10]). However, in proliferating VSMCs, whilst L-type Ca^2+^ channel expression decreases, T-type Ca^2+^ channel expression has long been known to increase [26, 42], and Ca^2+^ influx via T-type Ca^2+^ channels appears to be required for proliferation in vitro and neointima formation following vascular injury [26, 29, 43, 45]. The implication of a role for T-type Ca^2+^ channels has often been based on the use of mibefradil [29, 45], which is now known to exert effects on targets other than T-type Ca^2+^ channels (e.g. [15]). However, more recent molecular approaches have confirmed this class of channel as being of primary importance in vascular proliferation [43, 47].

Heme oxygenase (HO) enzymes catalyse the degradation of heme to biliverdin, Fe^2+^ and carbon monoxide (CO). Whilst HO-2 is constitutively active and widely distributed, HO-1 is induced by a variety of cellular stresses [23, 44] and regarded as protective, since heme itself is pro-oxidant, and biliverdin is rapidly converted to the powerful antioxidant, bilirubin. HO-1 induction affords protection in a variety of pathological cardiovascular conditions including myocardial infarction, hypertension, atherosclerosis and vascular injury, many of which involve VSMC proliferation [8, 44]. Indeed, HO-1 is established as being anti-proliferative, and CO may account for many of the effects of HO-1 in VSMCs [12, 13, 34]: inhalation of CO can inhibit the proliferation of VSMCs in intimal hyperplasia following vessel grafting [34, 41] and CO inhalation, as well as CO-releasing molecules (CORMs), are being developed for future cardiovascular therapy [14] despite the detailed mechanisms underlying its anti-proliferative effect remaining unknown. In recent years, we and others have suggested that specific ion channels are targets of regulation by CO and that their modulation may account for some of the important actions of CO [11, 22, 46, 53].

We have most recently demonstrated that recombinant and native neuronal T-type Ca^2+^ channels are inhibited by CO [5]. In the present study, we have investigated the potential role of T-type Ca^2+^ channel regulation by CO on cellular proliferation.

## Methods

### Cell culture

#### A7r5 cells

A7r5 cells (a smooth muscle cell line derived from rat thoracic aorta [24]) were obtained from the European Collection of Cell Cultures (ECACC, Public Health England, Porton Down, UK). They were grown in A7r5 complete media, consisting of Dulbecco's Modified Eagle Medium (DMEM) containing 10 % foetal bovine serum (FBS) (Biosera, Ringmer, UK) and 1 % glutamax (Gibco, Paisley, UK). Cells were kept in a humidified incubator at 37 °C (95 % air: 5 % CO_2_) and passaged weekly.

#### Human saphenous vein smooth muscle cells (HSVSMCs)

Smooth muscle cells were isolated from the saphenous vein (SV) of anonymous patients undergoing coronary bypass graft surgery at Leeds General Infirmary following ethical approval and informed patient consent. Segments of SV, around 1 cm in length, were denuded of endothelium and adventitia and were cut open longitudinally, lumen facing upwards. The segment was then divided into two pieces. Two milliliters of complete medium (DMEM containing 10 % (*v*/*v*) FBS (Biosera, Ringmer, UK), 1 % (*v*/*v*) L-glutamine and 1 % (*v*/*v*) penicillin/streptomycin (Gibco, Paisley, UK; unless otherwise stated)) were transferred into a clean petri dish and a segment of vein placed in the media. This was then cut with a razorblade into fragments around 0.5 mm^2^ in size. This tissue and media mixture was then transferred to a 25-cm^2^ tissue culture flask and maintained in a humidified atmosphere (37 °C; 95 % air: 5 % CO_2_). Cells migrated out from these tissue fragments within 7–10 days, and when 80–90 % confluent, the cells were plated for experimentation. HSVSMCs were used at passages between P1 and P6.

#### HEK293 cells

Wild type (WT; untransfected) HEK293 cells were cultured in minimum essential medium containing Earle’s salts and L-glutamine and supplemented with 10 % (*v*/*v*) foetal bovine serum (Biosera, Ringmer, UK), 1 % (*v*/*v*) non-essential amino acids, 1 % (*v*/*v*) antibiotic/antimycotic and 0.1 % (*v*/*v*) gentamicin. HEK293 cells stably expressing Ca_v_3.2 T-type Ca^2+^ channels (a kind gift from Prof. E. Perez-Reyes; University of Virginia, VA, USA) were cultured in WT HEK293 media, additionally supplemented with 1 mg/ml G-418 to maintain selection pressure (all reagents from Gibco, Paisley, UK; unless otherwise stated). HEK293/Ca_v_3.2 cells were used at passages between P1 and P8, and WT HEK293 cells were used at passages between P1 and P12; both cell types were kept in a humidified incubator at 37 °C (95 % air: 5 % CO_2_) and passaged weekly.

### Proliferation assay

Cells were plated in 24-well plates in complete media at 1 × 10^4^ cells per well. HSVSMCs were allowed to adhere overnight and subjected to serum free media (SFM) for 2.5 days. A7r5 and HEK293 cells were allowed to adhere for 6 h and then subjected to SFM overnight. On day 0 of the assay, SFM was removed and 1 ml of the relevant complete media was added to each well, in addition to the required drug or compound being investigated. To count cells, media was removed, cells were washed with 1 ml of Dulbecco’s phosphate buffered saline (PBS) and 200 μl of 0.05 % trypsin-EDTA (Gibco, Paisley, UK) was added (pre-warmed to 37 °C). Post-incubation, 800 μl of complete media was added and the cell suspension centrifuged (600*g* for 6 min). Following removal of 950 μl of media, 50 μl of supernatant remained with the cell pellet, which was then re-suspended with 50 μl of 0.4 % trypan blue (Thermo Scientific, Rockford, USA) to exclude unviable cells. Media was retained from one well of each treatment, processed in the same manner as the cell samples, and any cells present were counted as an additional quantification of non-viable cells. Day 0 counts and media counts were performed using a hemocytometer. All other counts were performed using a TC10 automated cell counter (Bio-Rad, Hemel Hempstead, UK).

### Western blotting

HSVSMCs, WT HEK293 and HEK293/Ca_v_3.2 cells were grown to 80 % confluence in 6-well plates. The wells were replenished with 0.4 % serum-containing media plus the required concentration of cobalt protoporphyrin IX (CoPPIX). Post-treatment, the cells were washed with PBS and lysed via incubation for 30 min with 200 μl mammalian protein extraction reagent (M-PER^TM^; Thermo Scientific, Rockford, USA) containing complete mini protease inhibitors (Roche Diagnostics Ltd., Lewes, UK). Cell lysates were retrieved and protein levels determined using a BCA protein assay kit according to manufacturers’ instructions (Thermo Scientific, Rockford, USA). Protein (10–20 μg) containing 2× sample buffer (250 mM Tris/HCl, pH 6.8, 4 % (*w*/*v*) SDS, 20 % (*w*/*v*) glycerol, 1 % bromophenol blue and 10 % β-mercaptoethanol) was loaded onto 12.5 %, 0.75-mm-thick polyacrylamide-sodium dodecyl sulphate gels and separated for ~1 h at 35 mA before being transferred onto 0.2 μm polyvinyl difluoride membranes at 30 V overnight. Membranes were blocked using 5 % (*w*/*v*) non-fat dried milk powder in tris buffered saline (TBS)-tween (0.05 %) for 1 h, then incubated with rabbit anti-HO-1 antibody raised against amino acids 184–288 of human HO-1 (SC-10789; Santa Cruz, Dallas, USA) at 1:200 for 3 h at room temperature (21–24 °C). Mouse anti-β-actin raised against the N-terminal of β-actin (Sigma, Gillingham, UK) was used as a loading control at 1:4,000. The membranes were then washed in TBS-tween (0.05 %) and incubated with the corresponding anti-rabbit or anti-mouse peroxidase-conjugated secondary antibody (GE Healthcare, Amersham, UK) at 1:2,000 for 1 h at room temperature. Protein bands were detected using the enhanced chemi-luminescent method (GE Healthcare, Amersham, UK) on hyperfilm. Densitometric analysis was performed using Image J (NIH UK).

### Electrophysiology

Ca^2+^ currents were recorded from A7r5 cells using the whole-cell configuration of the patch-clamp technique at room temperature (21–24 °C) as previously described [5] using an Axopatch 200A amplifier/Digidata 1300 interface controlled by Clampex 9.0 software (Molecular Devices, Sunnyvale, CA, USA). Offline analysis was performed using Clampfit 9.0. Pipettes (4–6 MΩ) were filled with (in mM) the following: CsCl 120, MgCl_2_ 2, EGTA 10, TEA-Cl 20, HEPES 10, Na-ATP 2 and pH 7.2 (adjusted with CsOH). To optimise recording of T-type Ca^2+^ currents, cells were perfused with (in mM) the following: NaCl 95, CsCl 5, MgCl_2_ 0.6, CaCl_2_ 15, TEA-Cl 20, HEPES 5, D-glucose 10 and pH 7.4 (adjusted with NaOH). Cells were voltage-clamped at −80 mV and either repeatedly depolarized to −20 mV (200 ms, 0.1 Hz) or to a series of test potentials ranging from −100 to +60 mV. To record L-type Ca^2+^ currents, extracellular Ca^2+^ was replaced with 20 mM Ba^2+^ (pH 7.4, adjusted with NaOH) and a holding potential of −50 mV was employed in order to inactivate T-type Ca^2+^ channels. Cells were repeatedly depolarized to +10 mV (200 ms, 0.1 Hz). All currents were low-pass filtered at 2 kHz and digitised at 10 kHz.

### Real-time polymerase chain reaction (RT-PCR)

To determine mRNA expression levels of Ca_v_3.2 and Ca_v_3.1 channels, T75 flasks (70–80 % confluency) were washed with PBS and cells dissociated using 0.5 ml 0.05 % trypsin-EDTA for 3 min (37 °C; 95 % air: 5 % CO_2_). Enzyme activity was halted by adding 0.5-ml ice-cold PBS; the cell suspension was then centrifuged (600*g* for 6 min). RNA was generated from whole cell lysates using the Aurum total RNA mini kit (Bio-Rad, Hemel Hempstead, UK) following manufacturer’s instructions. A cDNA template was generated from RNA samples using the iScript cDNA synthesis kit (Bio-Rad, Hemel Hempstead, UK) following manufacturer’s instructions (reaction profile was 5 min at 25 °C, 30 min at 42 °C, 5 min at 85 °C, 5 min at 4 °C). Rat or human Taqman probes (Applied Biosystems (ABI), UK) for Ca_v_3.1 (CACNA1G), Ca_v_3.2 (CACNA1H) and the endogenous housekeeper hypoxanthine phosphoribosyltransferase (HPRT1) were employed for A7r5 cells and HSVSMC, respectively. In all cases, 2 μl of sample cDNA and 18 μl of RT-PCR reaction mix (10 μl Taqman universal PCR master mix, 0.5 μl Taqman probes (both from ABI) and 7.5 μl RNase/DNase-free water (Gibco, Cambridge, UK)) were added to the required wells of a 96-well PCR plate (Applied Biosystems, Cambridge, UK). RT-PCR was carried out using an ABI 7500 real-time PCR system (reaction profile was 2 min at 50 °C, 10 min at 95 °C, 15 s at 95 °C for 60 cycles, 1 min at 60 °C). Data were analysed using the 7500 software (ABI) and relative gene expression calculated using the 2^−ΔΔCT^ method with HPRT1 as the endogenous control.

### Ca^2+^ microfluorimetry

WT HEK293 or HEK293/Ca_v_3.2 cells were plated at the required cell density on circular glass coverslips (10 mm, thickness 0) and allowed to adhere overnight. Cells were washed and incubated with 4 μM Fura 2-AM (Invitrogen, Cambridge, UK) diluted in HEPES-buffered saline for 40 min at room temperature (21–24 °C). Composition of HEPES-buffered saline was (in mM): NaCl 135, KCl 5, MgSO_4_ 1.2, CaCl_2_ 2.5, HEPES 5, glucose 10, osmolarity adjusted to 300 mOsm with sucrose, and pH adjusted to 7.4. The Fura 2-containing saline was removed after 40 min and replaced with HEPES-buffered saline for 15 min to allow de-esterification. Coverslip fragments were loaded into a perfusion chamber on an inverted epifluorescence microscope, and the cells were superfused via gravity at 2–3 ml/min. [Ca^2+^]_i_ was indicated by fluorescence emission measured at 510 nm as a result of alternating excitation at 340 and 380 nm using a Cairn Research ME-SE Photometry system (Cairn Research, Cambridge, UK). Baseline readings were obtained on exposure to HEPES-buffered saline, and Ca^2+^ homeostasis was monitored in response to the addition of a drug, or in response to Ca^2+^-free HEPES-buffered saline (composition as above, but with CaCl_2_ replaced by 1 mM EGTA).

Statistical comparisons were made using, as appropriate, paired or unpaired student’s *t* tests, one-way ANOVA with a multiple comparison test or repeated measures one-way ANOVA with a multiple comparison test.

## Results

### CO regulation of T-type Ca^2+^ channels regulates proliferation in A7r5 cells

**Fig. 1:**
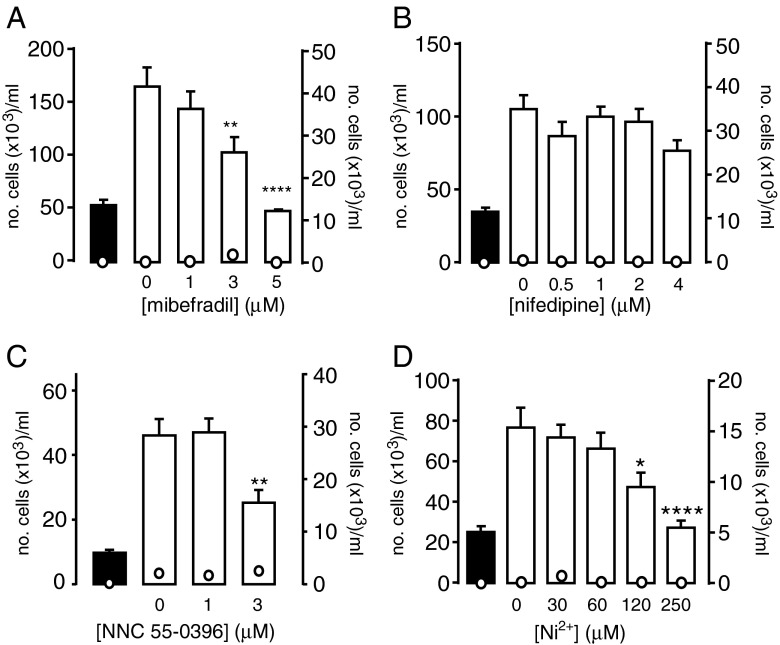
T-type Ca^2+^ channel inhibitors suppress proliferation of A7r5 cells. **a–d** *Bar graphs* showing the proliferative response (mean ± s.e.m) of A7r5 cells to increasing concentrations of specified drugs. Proliferation (plotted as *bar graphs*, corresponding to the left-hand *y*-axis) was monitored on day 0 (*solid bars*) and on day 3 (*open bars*) in the absence or presence of mibefradil (**a** *n* = 4), nifedipine (**b** *n* = 3), NNC 55-0396 (**c** *n* = 7) or Ni^2+^ (**d** *n* = 3, in the presence of 2 μM nifedipine throughout). The *open circles* show the corresponding non-viable cell count (plotted against corresponding right-hand *y*-axis). Statistical significance **p < 0.01, ****p < 0.0001 vs day 3 control (no drug). Data analysed via ratio repeated measures one-way ANOVA followed by Dunnett’s multiple comparison test

The known role of T-type Ca^2+^ channels in proliferation (see “Introduction”), together with our recent study indicating that CO can directly modulate T-type Ca^2+^ channels [5], indicates that HO-1-derived CO can limit proliferation via inhibition of T-type Ca^2+^ channels. To investigate this, we employed A7r5 cells, which are derived from rat aortic smooth muscle [24] and express T-type Ca^2+^ channels as well as L-type Ca^2+^ channels [6, 30, 39]. Mibefradil caused a concentration-dependent decrease in proliferation, as determined after 3 days, without loss of cell viability (Fig. 1a). By contrast, nifedipine did not significantly affect proliferation over the same time period at concentrations up to 4 μM (Fig. 1b). A previous electrophysiological study indicated that at 1 μM mibefradil was selective for T-type over L-type Ca^2+^ channels in A7r5 cells [6], but did not explore higher concentrations. Therefore, to probe the role of T-type Ca^2+^ channels in proliferation further, we also found that an alternative and more selective T-type Ca^2+^ channel blocker, NNC-55-0396 [20], significantly reduced proliferation at 3 μM (Fig. 1c), but was toxic to cells at higher concentrations (not shown). Finally, we investigated the effects of Ni^2+^, a known T-type Ca^2+^ channel inhibitor. Importantly, these studies were performed in the presence of 2 μM nifedipine in order to prevent any potential influence of L-type Ca^2+^ channel blockade by Ni^2+^ on proliferative responses. Ni^2+^ caused a concentration-dependent inhibition of proliferation, as shown in Fig. 1d. The data presented in Fig. 1 strongly suggest that Ca^2+^ influx via T-type, but not L-type Ca^2+^ channels, contributes to the proliferation of A7r5 cells.

**Fig. 2:**
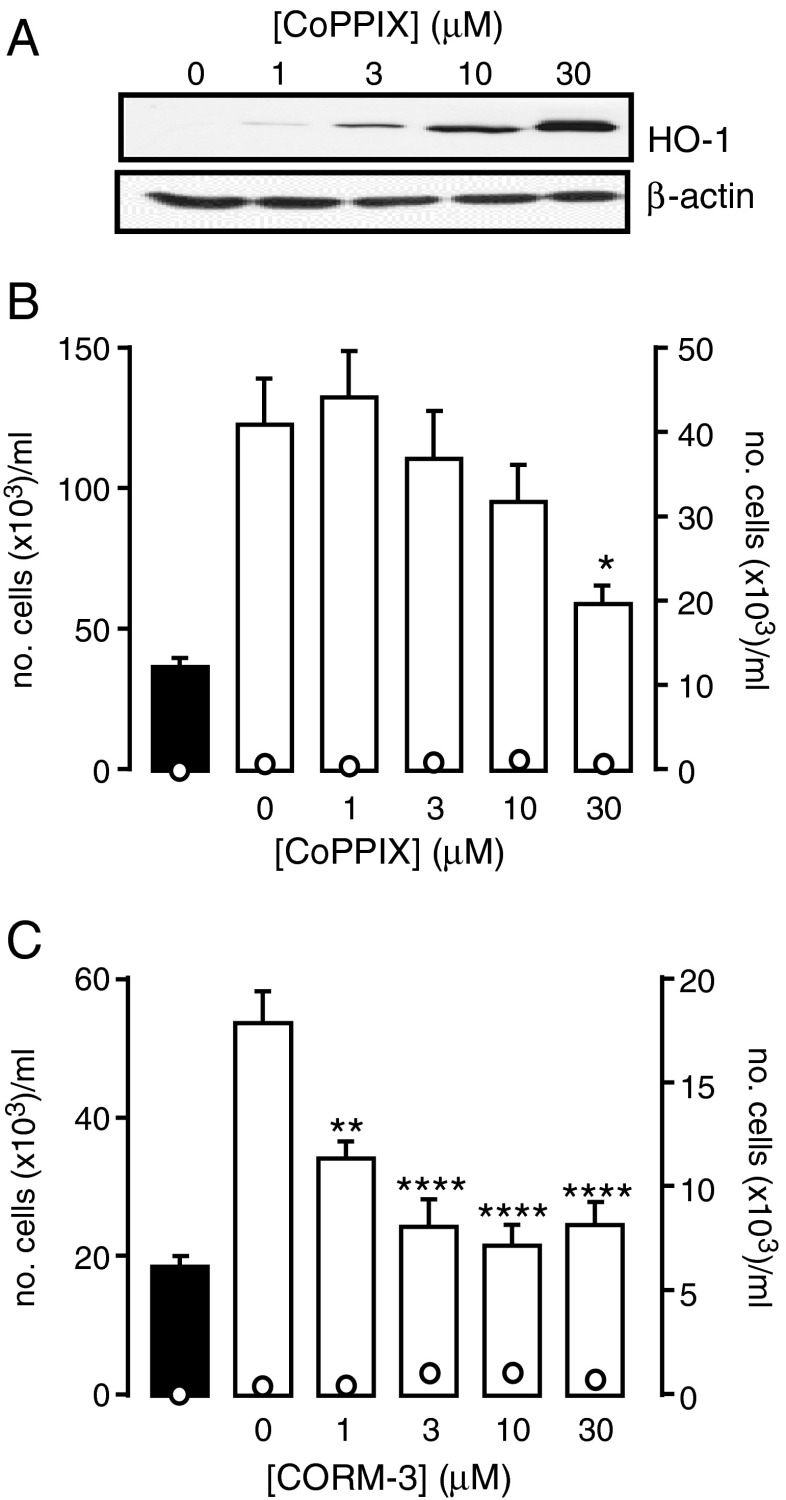
HO-1 and CO inhibit proliferation in A7r5 cells. **a** Western blot showing the concentration-dependent induction of HO-1 expression by CoPPIX in A7r5 cells. Corresponding β-actin blots are shown below. **b** *Bar graph* showing the proliferative response (mean ± s.e.m, *n* = 5) of A7r5 cells following HO-1 induction. Proliferation (plotted as *bars*, corresponding to the left-hand *y*-axis) was monitored on day 0 (*solid bar*) and on day 3 (*open bars*) in the absence or presence of CoPPIX to induce HO-1. The *open circles* show the corresponding unviable cell count (plotted against corresponding right-hand *y*-axis). Statistical significance *p < 0.05 day 3 control (no drug). **c** *Bar graph* showing the proliferative response (mean ± s.e.m, *n* = 5) of A7r5 cells in the absence or presence of increasing concentrations of CORM-3. Proliferation (plotted as *bars*, corresponding to the left-hand *y*-axis) was monitored on day 0 (*solid bar*) and on day 3 (*open bars*) in the absence or presence of CORM-3. The *open circles* show the corresponding unviable cell count (plotted against corresponding right-hand *y*-axis). Statistical significance **p < 0.01, ****p < 0.0001 vs day 3 control (no drug). Data analysed via one-way ANOVA (**a**) or ratio repeated measures one-way ANOVA (**b** and **c**) followed by Dunnett’s multiple comparison test

**Fig. 3:**
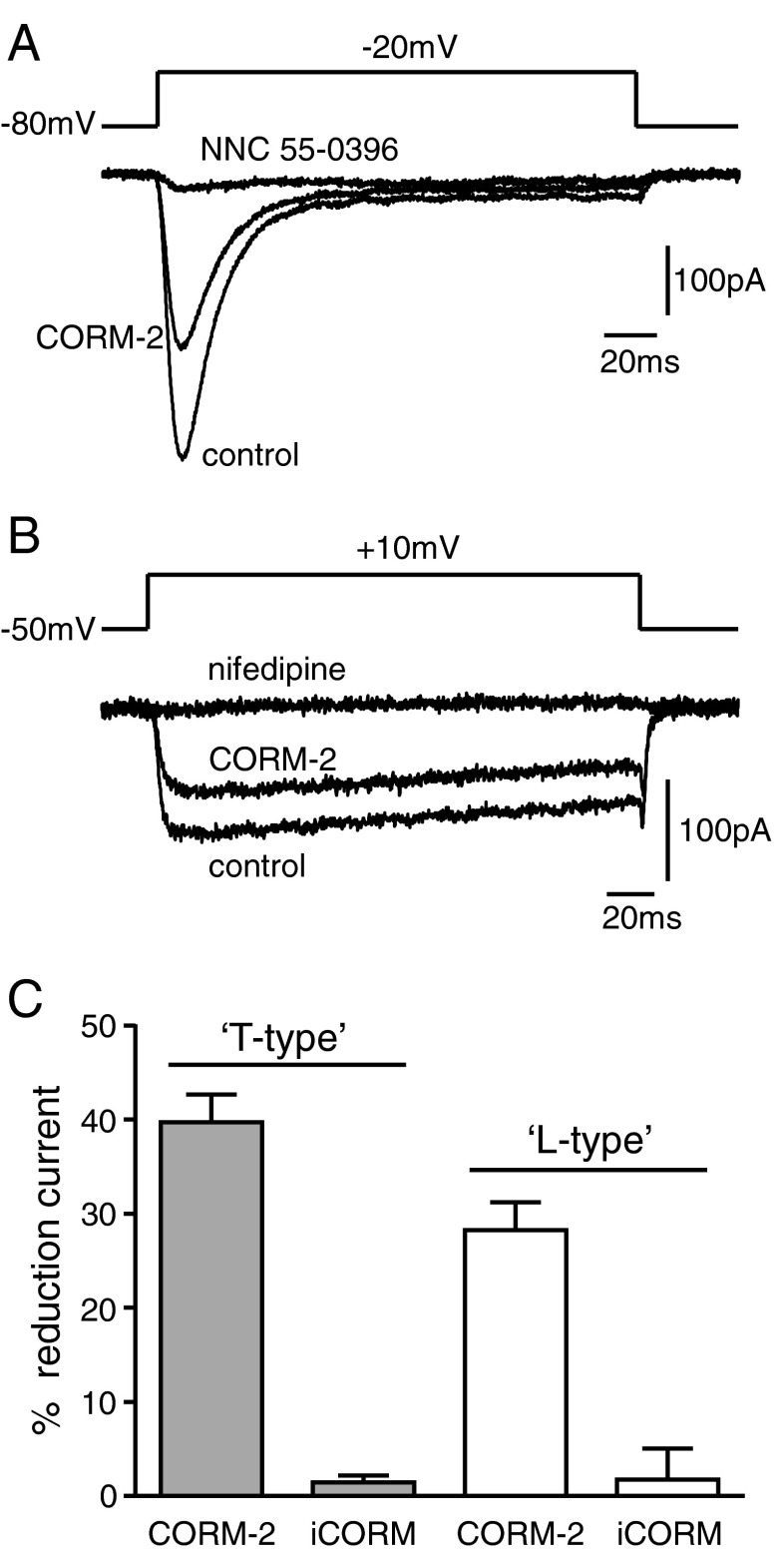
CO inhibits both T-type and L-type Ca^2+^ currents in A7r5 cells. **a** Example currents evoked in A7r5 cells using the voltage command protocol indicated above. The cell was perfused with a control solution (containing Ca^2+^ as the charge carrier), then exposed to 3 μM CORM-2 and, following washout of CORM-2, 3 μM NNC 55-0396. Such transient currents recorded under these conditions were considered attributable to the activity of T-type Ca^2+^ channels. **b** as **a**, except that Ba^2+^ rather than Ca^2+^ was used as the charge carrier, and currents were evoked from a more depolarized holding potential, as indicated. Currents shown were evoked before (control) and during exposure to 3 μM CORM-2 and, following washout of CORM-2, 2 μM nifedipine, as indicated. Such sustained currents recorded under these conditions were considered attributable to the activity of L-type Ca^2+^ channels **c** *Bar graph* showing mean inhibition of T-type Ca^2+^ currents (*shaded bars*, recorded as in **a**, *n* = 11 cells) and L-type Ca^2+^ currents (*open bars*, recorded as in **b**, *n* = 12) caused by 3 μM CORM-2. Effects of 3 μM iCORM (*n* = 5 for each) are also indicated

Exposure of A7r5 cells to CoPPIX caused a concentration-dependent increase in the expression of HO-1, as detected by Western blotting (Fig. 2a). This procedure for induction of HO-1 caused a significant reduction of proliferation in A7r5 cells (Fig. 2b). Furthermore, proliferation of A7r5 cells was strikingly reduced by exposure of cells to CORM-3 (Fig. 2c). Collectively, the data presented in Figs. 1 and 2 suggest that proliferation in A7r5 cells is dependent on T-type Ca^2+^ channel activity and can be inhibited by induction of HO-1 or exposure to CO. To investigate whether CO acted via inhibition of native T-type Ca^2+^ channels in these cells, we examined their activity using whole-cell patch-clamp recordings. T-type Ca^2+^ channel currents, recorded using a holding potential of −80 mV and Ca^2+^ as the charge carrier, were inhibited by exposure of cells to CORM-2 but not to iCORM (Fig. 3a, c). Where tested (e.g. Fig. 3a), these currents were also inhibited by 3 μM NNC 55-0396 (93.2 ± 5.9 % inhibition, *n* = 5). To study L-type Ca^2+^ currents, we used a holding potential of −50 mV (in order to inactivate T-type Ca^2+^ channels) and replaced Ca^2+^ with Ba^2+^ to promote influx via L-type rather than T-type Ca^2+^ channels. Under these conditions, currents displaying little or no inactivation were also inhibited by CORM-2 but not iCORM (Fig. 3b, c) and, where tested (e.g. Fig. 3b), were inhibited by 2 μM nifedipine (88.5 ± 6.2 % inhibition, *n* = 5). Thus, CO can inhibit both T-type and L-type Ca^2+^ channels natively expressed in A7r5 cells.

### HO-1 and CO inhibit proliferation in HSVSMCs

**Fig. 4:**
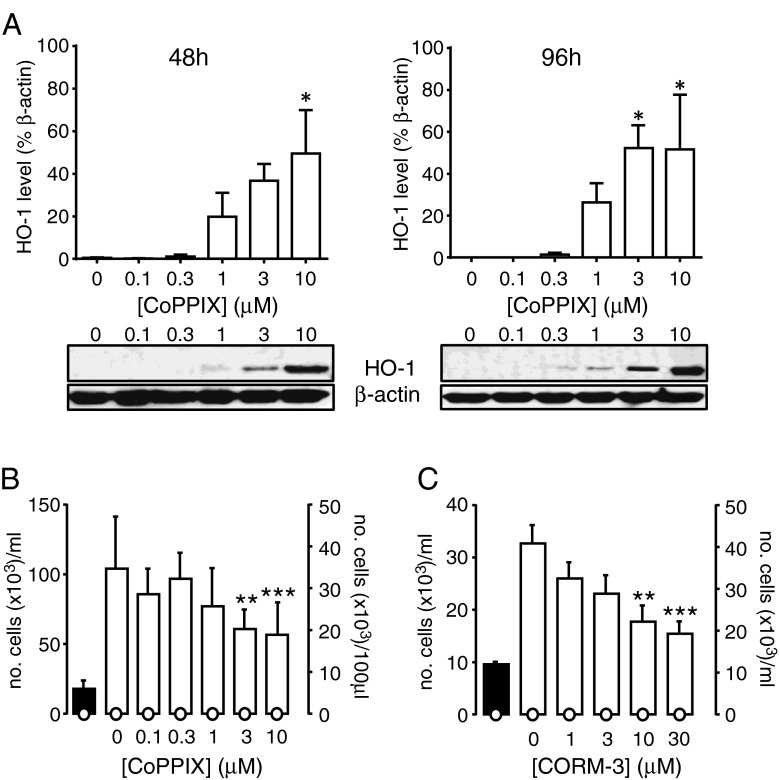
HO-1 and CO inhibit proliferation in human saphenous vein SMCs. **a** *Bar graphs* showing the relative HO-1 protein expression in HSVSMCs following 48 h (*left*) and 96 h (*right*) exposure to CoPPIX at the concentrations indicated; densitometric analyses were normalised to β-actin (*n* = 3 in each case). CoPPIX treatment was added at 0 and 48 h. Data are represented as mean ± s.e.m., and data were analysed by one-way ANOVA with Dunnett’s multiple comparison test; statistical significance *p < 0.05 vs control levels. Representative Western blots of HO-1 and the corresponding β-actin loading control at 48 and 96 h are shown below. **b** *Bar graph* showing the proliferative response of HSVSMC (plotted against corresponding left *y*-axis) to increasing concentrations of CoPPIX. The *open circles* show the corresponding unviable cell count (plotted against corresponding right *y*-axis). Statistical significance **p < 0.01, ***p < 0.001 vs day 3 control (no CoPPIX). Data are represented as mean ± s.e.m. (*n* = 4). **c** *Bar graph* showing the proliferative response of HSVSMC (plotted against corresponding left *y*-axis) to increasing concentrations of CORM-3. The *open circles* show the corresponding unviable cell count (plotted against corresponding right *y*-axis). Statistical significance **p < 0.01, ***p < 0.001 vs day 3 control (no CORM-3). Data are represented as mean ± s.e.m. (*n* = 4). Data analysed via one-way ANOVA (**a**), or ratio repeated measures one-way ANOVA followed by Dunnett’s multiple comparison test (**b** and **c**)

To examine whether the HO-1/CO pathway was able to modify proliferation in human VSMCs, we studied cells cultured from human saphenous vein. Figure 4a shows that HO-1 could be induced in these cells in a concentration-dependent manner and that induction was clearly detectable at 2 and 4 days (the duration of associated proliferation studies). Induction of HO-1 also led to a concentration-dependent inhibition of proliferation over this same time period, without loss of cell viability (Fig. 4b). To investigate whether the reduced proliferation observed following HO-1 induction was attributable to the production of CO, we exposed cells to CORM-3 and found that this agent caused a concentration-dependent inhibition of proliferation, again without any loss of cell viability (Fig. 4c).

**Fig. 5:**
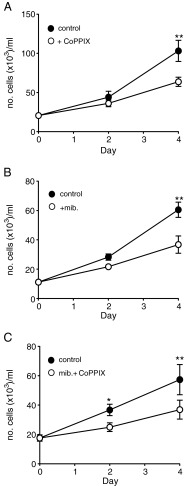
Mibefradil and HO-1 induction are non-additive in suppressing human saphenous vein SMC proliferation**. a–c** *Line graphs* showing proliferation of HSVSMCs monitored over a 4-day period, in the absence of drug treatment (*solid circles*), or during HO-1 induction with 3 μM CoPPIX (*open symbols*, **a**), or in the presence of 3 μM mibefradil (*open circles*, **b**), or during simultaneous application of 3 μM mibefradil and 3 μM CoPPIX (*open circles*, **c**). Each point represents mean ± s.e.m. (*n* = 5). Statistical significance *p < 0.05, **p < 0.01. Data analysed via repeated measures one-way ANOVA followed by Sidak’s multiple comparison test between control and treated groups for each timepoint

Figure 5a shows a proliferation time-course experiment from HSVSMCs, and again demonstrates the inhibitory effect of HO-1 induction, using 3 μM CoPPIX. A qualitatively and quantitatively similar effect was found when cells were exposed to the known T-type Ca^2+^ channel blocker, mibefradil (3 μM; Fig. 5b), which was without effect on cell viability (data not shown). Finally, proliferation was again reduced by a similar amount in cells in which HO-1 had been induced, and during an additional exposure to mibefradil (Fig. 5c), indicating that HO-1 and mibefradil are non-additive, likely because they act via the same target, the T-type Ca^2+^ channel.

**Fig. 6:**
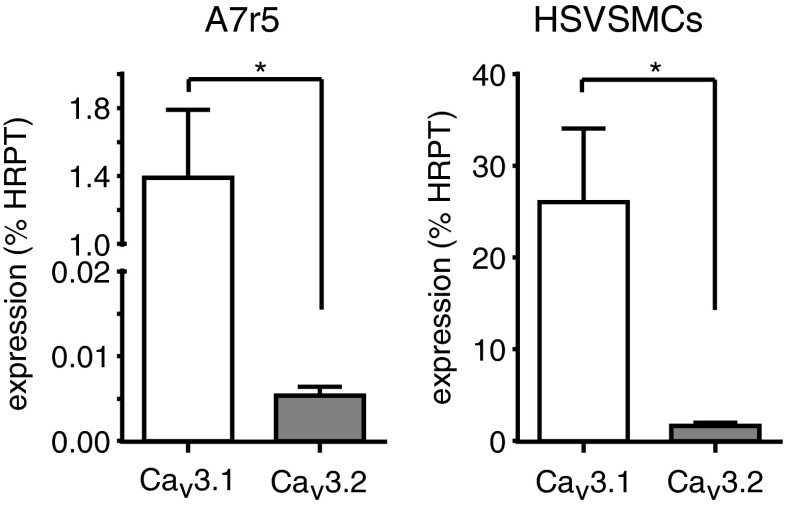
Expression levels for Ca_v_3.1 and Ca_v_3.2 mRNA determined in A7r5 cells and HSVSMCs, as indicated. Channel expression is plotted as mean ± s.e.m. percentage of expression of the housekeeping gene, hypoxanthine phosphoribosyltransferase (HPRT1), taken from 7 A7r5 samples and 6 HSVSMC samples. Statistical significance *p < 0.05, data analysed via unpaired *t* test

Figure 6 shows the expression levels, relative to the endogenous housekeeper HPRT1, of mRNA for the T-type Ca^2+^ channel isoforms, Ca_v_3.1 and Ca_v_3.2, as determined by RT-PCR. In both the A7r5 cells and HSVSMCs, the Ca_v_3.1 isoform is expressed at significantly higher levels than the Ca_v_3.2 isoform, but both isoforms were detected.

### CO inhibits augmented proliferation in Cav3.2-expressing HEK293 cells

**Fig. 7:**
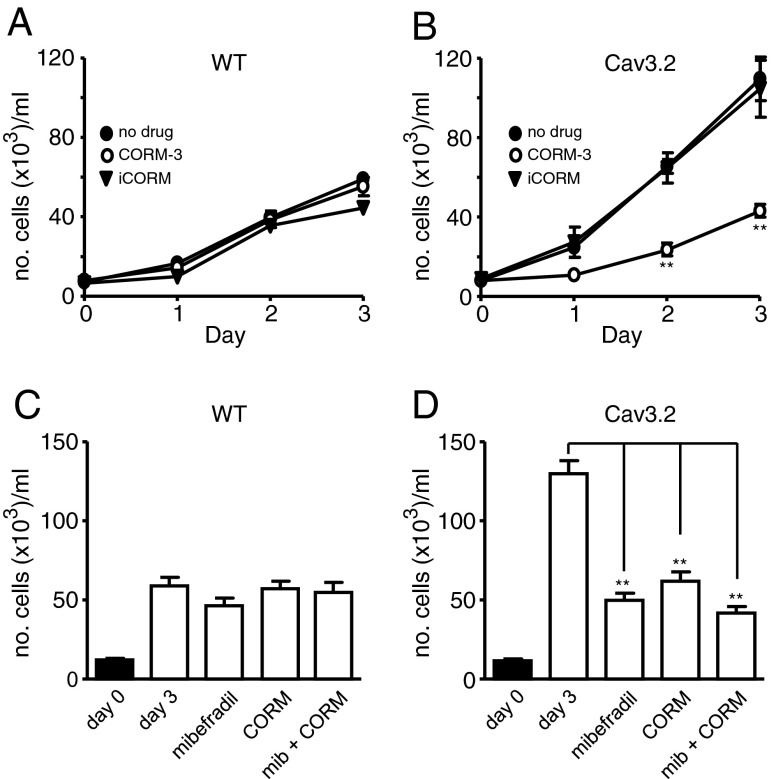
CO inhibits the augmented proliferation observed in Ca_v_3.2-expressing HEK293 cells. **a** and **b** Plots of mean (± s.e.m., *n* = 3) proliferation monitored in untransfected (wild type; WT) and Ca_v_3.2-expressing HEK293 cells, as indicated. Cells were cultured in the absence of drugs (*solid circles*), or in the presence of either CORM-3 (30 μM; *open circles*) or iCORM (30 μM *solid triangles*). **c** and **d** *Bar graphs* illustrating the effects of mibefradil and CORM-3 (applied separately or together, as indicated) on proliferation measured on day 3 in WT (**c**) and Ca_v_3.2-expressing HEK293 cells (**d**). Each *bar* represents mean (± s.e.m.) proliferation determined from 9 repeats. Statistical significance: **P < 0.01 as compared with controls. Data analysed via ratio repeated measures one-way ANOVA followed by Dunnett’s multiple comparison test

In order to better understand the cellular mechanisms underlying CO modulation of T-type Ca^2+^ channels and how this impacts on proliferation, we employed a recombinant expression system. Preliminary studies in HEK293 cells stably expressing Ca_v_3.1 indicated that these cells readily formed clumps and became detached in culture, making assessment of their effects on proliferation difficult. We therefore focussed on cells over-expressing Ca_v_3.2, which are also expressed in VSMCs (see [49] as well as Fig. 6), and are equally potently modulated by CO [5]. In agreement with a previous report [17], we found that over-expression of Ca_v_3.2 in HEK293 cells increased their proliferation when compared with WT cells over a 3-day period (Fig. 7a, b). Exposure of WT cells to the CO-releasing molecule CORM-3 (30 μM) or the inactive, control compound iCORM (30 μM) was without significant effect on proliferation (Fig. 7a). By contrast, exposure of Ca_v_3.2-expressing cells to 30 μM CORM-3 (but not iCORM) significantly reduced proliferation (Fig. 7b). Proliferation monitored after 3 days also revealed that mibefradil (3 μM) was without significant effect in WT cells (Fig. 7c), but reduced proliferation in Ca_v_3.2-expressing cells to levels observed in WT cells, and CORM-3 was without further effect in the presence of mibefradil (Fig. 7d).

### Ca_v_3.2 over-expression increases basal [Ca^2+^]_i_

**Fig. 8:**
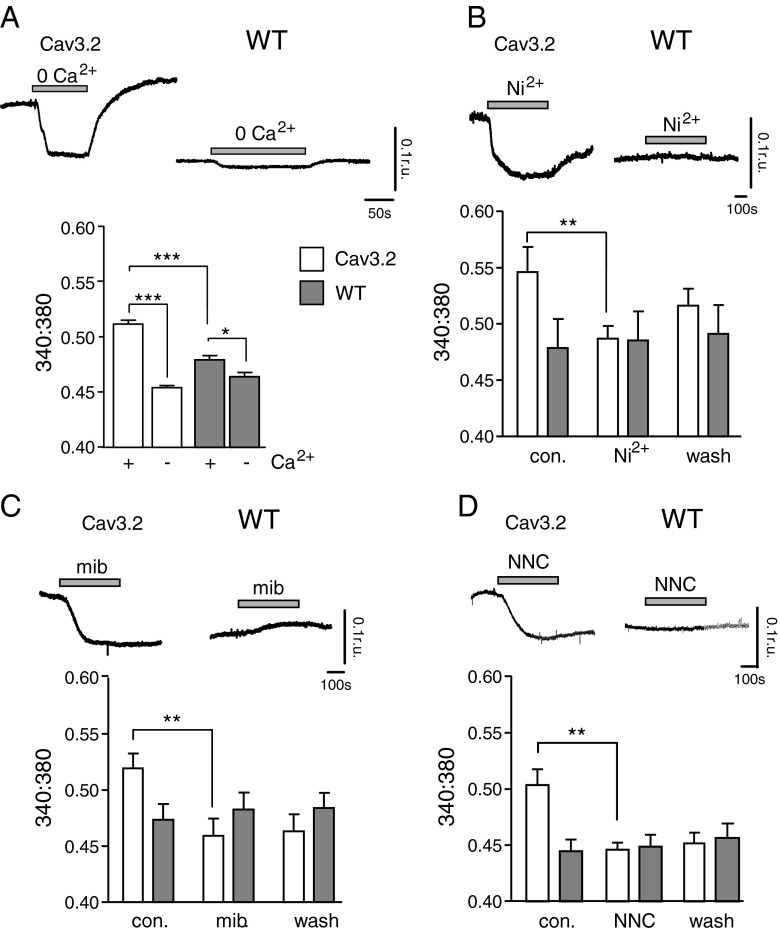
T-type Ca^2+^ channels influence basal [Ca^2+^]_i_ in Ca_v_3.2-expressing HEK293 cells. **a** *Upper traces* show examples of basal [Ca^2+^]_i_ recorded in Ca_v_3.2-expressing and untransfected (wild type; WT) HEK293 cells, as annotated. For the periods indicated by the *horizontal bars*, extracellular Ca^2+^ was replaced with 1 mM EGTA. *Below*; *bar graph* illustrating the mean basal [Ca^2+^]_i_ levels (with s.e.m. *bars*) recorded in Ca_v_3.2-expressing cells (*open bars*, *n* = 6) and WT cells (*shaded bars*, *n* = 6) in the presence and absence of extracellular Ca^2+^, as indicated. **b** *Upper traces* show examples of basal [Ca^2+^]_i_ recorded in Ca_v_3.2-expressing and WT HEK293 cells and the effects of Ni^2+^ (30 μM), applied for the periods indicated by the *horizontal bars. Below*; *bar graph* illustrating the mean (± s.e.m.) basal [Ca^2+^]_i_ levels recorded in Ca_v_3.2-expressing cells (*open bars*, *n* = 6) and WT cells (*shaded bars*, *n* = 6) before (con.), during (Ni^2+^) and after (wash) exposure to Ni^2+^, as indicated. **c** and **d** as **b**, except that cells were exposed to 3 μM mibefradil (mib; **c**) or 3 μM NNC55-0396 (NNC; **d**) for the periods indicated by the *horizontal bars*. Corresponding *bar graphs* illustrate mean (± s.e.m.) basal [Ca^2+^]_i_ levels recorded in Ca_v_3.2-expressing cells and WT cells before (con.), during (mib or NNC) and after (wash) exposure to mibefradil (**c** *n* = 7) or NNC (**d** *n* = 8), as indicated. Statistical significance *P < 0.05; **P < 0.01, ***P < 0.001 as compared with appropriate controls. Data analysed via paired or unpaired *t* test as appropriate

Tonic Ca^2+^ entry via the window current generated in cells expressing T-type Ca^2+^ channels is believed to regulate cell proliferation (see “Introduction”). We employed fluorimetric recordings from Fura-2 loaded HEK293 cells to both monitor Ca^2+^ levels and determine how they were influenced by T-type Ca^2+^ channel expression. Basal [Ca^2+^]_i_ in HEK293 cells expressing Ca_v_3.2 was significantly higher than levels observed in WT cells, and removal of extracellular Ca^2+^ (replaced with 1 mM EGTA) caused a fall of [Ca^2+^]_i_ which was far larger than that seen in WT cells (although the same manoeuvre also caused a significant decrease of [Ca^2+^]_i_ in these cells; Fig. 8a), in agreement with an earlier report [9]. To determine whether the elevated [Ca^2+^]_i_ was attributable to Ca^2+^ influx via the T-type Ca^2+^ channel window current, we investigated the effects of the T-type Ca^2+^ channel blockers Ni^2+^ (30 μM; Fig. 8b), mibefradil (3 μM; Fig. 8c) and NNC55-0396 (3 μM; Fig. 8d). All blockers caused significant reductions in [Ca^2+^]_i_, and in the case of Ni^2+^, this effect was at least partly reversible. None of the inhibitors tested significantly altered [Ca^2+^]_i_ in WT cells (Fig. 8b–d).

### HO-1 and CO regulate [Ca^2+^]_i_ in Ca_v_3.2-expressing cells

**Fig. 9:**
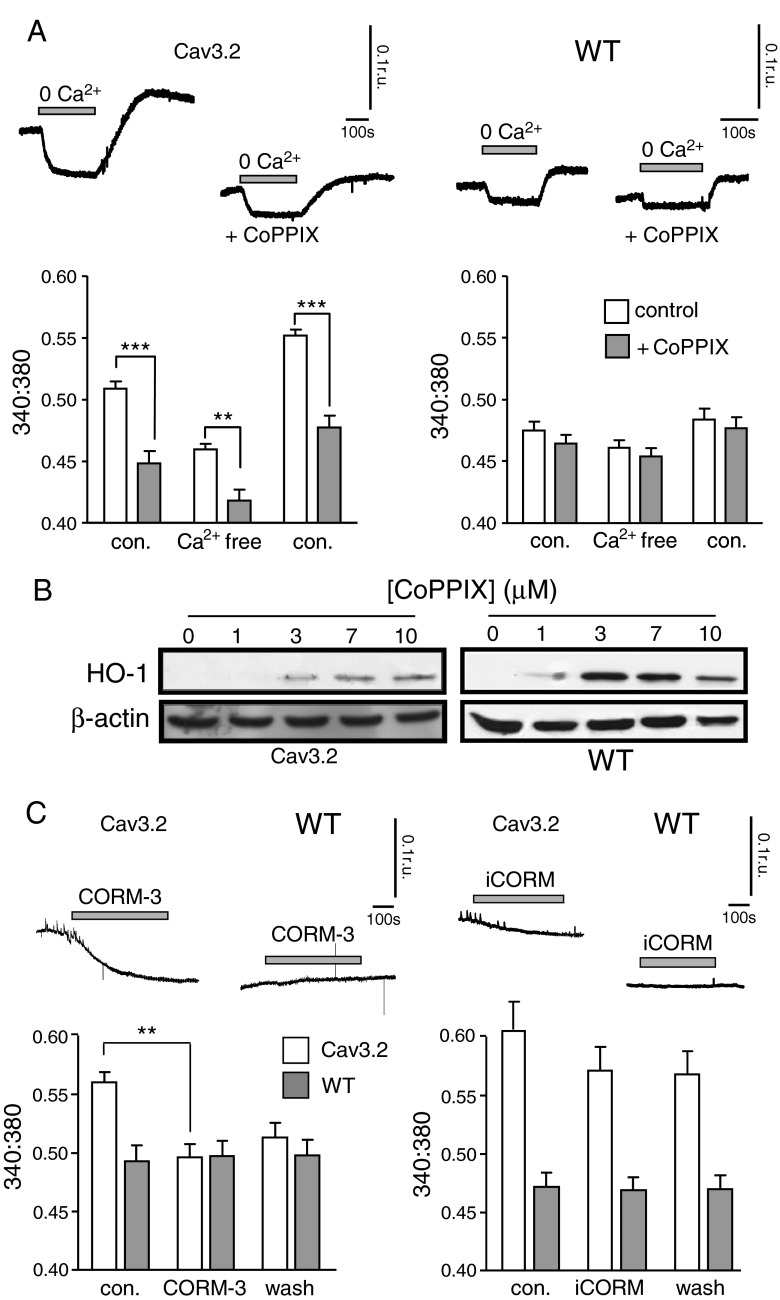
HO-1 and CO modulate basal [Ca^2+^]_i_ in Ca_v_3.2-expressing HEK293 cells. **a** *Upper traces* show examples of basal [Ca^2+^]_i_ recorded in Ca_v_3.2-expressing cells (*left traces* and *bar graph*) and WT cells (*right traces* and *bar graph*). Cells received either no pre-treatment, or were exposed to 10 μM CoPPIX (Ca_v_3.2) or 3 μM CoPPIX (WT) for 48 h to induce HO-1 expression (+CoPPIX). For the periods indicated by the *horizontal bars*, extracellular Ca^2+^ was replaced with 1 mM EGTA. *Below*; *Bar graphs* illustrating the mean (± s.e.m.) basal [Ca^2+^]_i_ levels recorded in Ca_v_3.2-expressing cells (*left bar graph*, *n* = 16) and WT cells (*right bar graph*, *n* = 12) before (con.), during (Ca^2+^ free) and after (con.) removal of extracellular Ca^2+^. *Open bars*; control cells. *Shaded bars*; exposed to 10 μM CoPPIX (Ca_v_3.2) or 3 μM CoPPIX (WT) for 48 h to induce HO-1 expression (+CoPPIX). Statistical significance **P < 0.01, ***P < 0.001 as compared with appropriate controls. **b** Western blots showing the concentration-dependent induction of HO-1 expression by CoPPIX. Corresponding β-actin blots are shown below, and data were obtained in Ca_v_3.2-expressing (*left*) and WT (*right*) HEK293 cells. **c** *Upper traces* show examples of basal [Ca^2+^]_i_ recorded in Ca_v_3.2-expressing and WT HEK293 cells, as indicated, and the effects of CORM-3 (3 μM; *left traces*) and iCORM (3 μM; *right traces*) applied for the periods indicated by the *horizontal bars. Below*; *bar graph* illustrating the mean (± s.e.m.) basal [Ca^2+^]_i_ levels recorded in Ca_v_ **P < 0.01 *P* < 0.001"?> as compared with appropriate controls. Data analysed via paired or unpaired *t* test as appropriate

We next investigated the effects of HO-1 induction on [Ca^2+^]_i_ in HEK293 cells. As illustrated in Fig. 9a, HO-1 induction with 10 μM cobalt protoporphyrin IX (CoPPIX) for 48 h caused a significant reduction in basal [Ca^2+^]_i_ in cells expressing Ca_v_3.2, although removal of extracellular Ca^2+^ reduced [Ca^2+^]_i_ further. By contrast, HO-1 induction with 3 μM CoPPIX in WT HEK293 cells was without significant effect (Fig. 9a). This slightly lower concentration of CoPPIX was selected for WT HEK293 cells, since it was found to be the optimal concentration for HO-1 induction, as determined by Western blotting, whereas in Ca_v_3.2-expressing cells, maximal induction was achieved with 10 μM CoPPIX (Fig. 9b). To determine whether CO mediated the effects of HO-1 induction on resting [Ca^2+^]_i_, we applied CORM-3 (3 μM), which caused a striking and largely irreversible reduction of [Ca^2+^]_i_ in Ca_v_3.2-expressing HEK293 cells, but not in WT cells (Fig. 9c). By contrast, iCORM was without significant effect in either cell type (Fig. 9c). Collectively, these fluorimetric studies indicate that over-expression of Ca_v_3.2 generates a detectable tonic Ca^2+^ influx in HEK293 cells which can be suppressed either by CO or following induction of HO-1.

## Discussion

Although Ca^2+^ influx via L-type Ca^2+^ channels is important for VSMC contraction, a reduction in their expression is associated with the proliferative phenotypic change [16, 19], as observed in pathological models involving VSMC proliferation [40]. However, Ca^2+^ influx is still needed for the progression of proliferation since it regulates the activity of numerous transcription factors, e.g. NFAT (nuclear factor of activated T-cells; [2]). Some studies suggest TRP (transient receptor potential) channels, particularly TRPC channels, contribute to Ca^2+^ influx during VSMC proliferation [19, 27]. Further evidence indicates STIM1/Orai–mediated Ca^2+^ entry is also involved in VSMC proliferation, migration and neointima formation in vivo [3, 56]. However, there is also compelling evidence for the involvement of voltage-gated T-type Ca^2+^ channels in VSMC proliferation. Indeed, in proliferating VSMCs, as L-type Ca^2+^ channel expression decreases, there is a concomitant increase in T-type Ca^2+^ channel expression [26, 42]. Evidence suggests Ca^2+^ influx via T-type Ca^2+^ channels is required for VSMC proliferation in vitro and in neointima formation observed following vascular injury [26, 29, 43, 45]. Although the implication of a role for T-type Ca^2+^ channels has often (but not always) been based on the use of mibefradil (which was originally proposed as a selective T-type Ca^2+^ channel blocker but has since been shown to exert other effects, such as inhibition of store-operated Ca^2+^ entry [15]), mibefradil clearly blocks T-type Ca^2+^ channels, inhibits proliferation associated with vascular injury-mediated neointima formation and NFAT-mediated transcriptional activity [29, 45]. Furthermore, in the pulmonary vasculature, evidence for T-type Ca^2+^ channels regulating proliferation comes also from siRNA-targeted T-type (Ca_v_3.1) Ca^2+^ channel knock-down [43]. Most convincingly, murine knockout models have recently shown beyond doubt that Ca_v_3.1 is required for VSMC proliferation following systemic vascular injury [47].

In VSMCs expressing native T-type Ca^2+^ channels (A7r5 cells and HSVSMCs), data presented are also consistent with these channels exerting an important influence on proliferation. Consistent with previous work [49], we detected expression of both Ca_v_3.1 and Ca_v_3.2 in A7r5 cells, and also detected mRNA for both channel types in HSVSMCs (Fig. 6), and mibefradil reduced proliferation in both cell types (Figs. 1 and 5). In A7r5 cells, despite the presence of nifedipine-sensitive L-type Ca^2+^ channels (Fig. 3), nifedipine was without effect on proliferation (Fig. 1), which discounts the possibility that mibefradil (or indeed NNC 55-0396) reduced proliferation via a non-selective blockade of L-type Ca^2+^ channels. Ni^2+^ (studied in the presence of nifedipine) was effective at reducing proliferation only at higher (>100 μM) concentrations. This suggests that influx of Ca^2+^ into A7r5 cells via T-type Ca^2+^ channels predominantly involves Ca_v_3.1 rather than Ca_v_3.2 channels, since Ca_v_0.3.2 channels would be expected to be already fully inhibited at these higher Ni^2+^ concentrations [28].

The major finding of the present study is that HO-1 induction leads to reduced proliferation in VSMCs (both A7r5 cells, Fig. 1, and HSVSMCs, Figs. 4 and 5) and that this occurs via CO formation which in turn inhibits T-type Ca^2+^ channels. Thus, reduced proliferation arising from HO-1 induction could be mimicked by application of the CO-donor CORM-3 in both cell types (Figs. 2 and 4), and in A7r5 cells, we were able to demonstrate directly that T-type Ca^2+^ channels were inhibited by CORM-2 (Fig. 3). It should be noted that we could not use CORM-2 for proliferation studies, since cells did not tolerate long-term exposure to its solvent, DMSO (data not shown). CO also inhibited L-type Ca^2+^ channels (as we have previously shown in cardiac myocytes [46]), but this appears to be without influence on proliferation, since proliferation was insensitive to nifedipine (Fig. 1b). The reason why L-type Ca^2+^ channels do not influence proliferation in these cells is unknown, but may be due to a lack of tonic activity at the cell’s resting membrane potential. In HSVSMCs, the lack of additive effects of HO-1 induction and mibefradil exposure on proliferation further support the idea that T-type Ca^2+^ channel modulation by CO accounts for the inhibition of proliferation by HO-1. These data, combined with our recent electrophysiological study directly demonstrating inhibition of all 3 isoforms of T-type Ca^2+^ channels by CO [5], and also the observation that HO-1 induction or exposure to CO reduces basal [Ca^2+^]_i_ in Ca_v_3.2-expressing cells and reduces proliferation, collectively argue strongly that VSMC proliferation can be regulated via T-type Ca^2+^ channel modulation by CO derived from HO-1.

T-type Ca^2+^ channels are also clearly associated with proliferation in other cell types, including certain cancers [37], where they represent viable therapeutic targets (e.g. [18]). The present study also demonstrates, in agreement with an earlier report [17], that over-expression of T-type Ca^2+^ channels (in this case, Ca_v_3.2; Fig. 7) in HEK293 cells promotes proliferation. This increase is attributable to Ca^2+^ influx via these channels, since inhibition with mibefradil reduced proliferation rates to levels observed in WT cells (i.e. not expressing T-type Ca^2+^ channels). Furthermore, Ca_v_3.2-mediated increases in proliferation were associated with increased basal [Ca^2+^]_i_ (Fig. 8), suggesting that tonic Ca^2+^ influx via Ca_v_3.2 provided a sustained elevation of [Ca^2+^]_i_ which promoted proliferation. This presumably occurs via the well-described T-type Ca^2+^ channel ‘window current’ [38] which arises from a small proportion of the total T-type Ca^2+^ channel population that retains tonic activity (i.e. partially activated and not fully inactivated) at or around the cell’s resting membrane potential. The presence of a window current generated by expressed Ca_v_3.2 channels would also require that native currents set the resting membrane potential appropriately. The nature of such currents has not to date been explored in depth.

Numerous studies have indicated that HO-1 induction affords protection in a variety of pathological systemic cardiovascular conditions, many of which involve VSMC proliferation [8, 44]. Indeed, HO-1 is established as being anti-proliferative, and its induction is observed in these proliferative vascular diseases. For example, proliferation associated with hypertension is suppressed by HO-1 induction [7], and many studies have indicated that HO-1 protects against inflammation and oxidative stress associated with atherosclerosis [1]. Furthermore, whilst bilirubin is a powerful antioxidant in its own right, evidence points towards CO as accounting for many of the effects of HO-1 in VSMCs [12, 13, 34]. For example, inhalation of CO inhibits VSMC proliferation in intimal hyperplasia following vessel grafting [34, 41] and CO inhalation, as well as CO-releasing molecules (CORMs), is being developed for future cardiovascular therapy [14, 32]. HO-1 is also influential in the pulmonary vasculature: hypoxic induction of pulmonary hypertension (and hence right ventricular dilation) was far worse in HO-1^−/−^ mice [55], and targeted pulmonary over-expression of HO-1 prevents inflammation and pulmonary vessel wall hypertrophy (including VSMC proliferation) in response to hypoxia [31]. Clearly, HO-1 is beneficial in providing protection against pulmonary remodelling, and speculation that CO mediates these effects of HO-1 has been made in earlier publications [25]. More recent studies have provided direct evidence that CO inhalation in HO-1^−/−^ mice prevented hypoxic pulmonary remodelling and inhibited hypoxic VSMC proliferation in vitro, whereas biliverdin was ineffective [51].

Despite these numerous accounts of the beneficial effects of CO derived from HO-1 induction in both systemic and pulmonary vascular diseases, no mechanism has been proposed to account for such effects to date. The data presented here provide compelling evidence that CO may provide protection against proliferative vascular diseases at least in part via inhibition of T-type Ca^2+^ channels. Since CO can regulate all three isoforms of T-type Ca^2+^ channels (Ca_v_3.1, 3.2 and 3.3 [5]), this regulation can occur regardless of which isoform is expressed. The present data therefore provide mechanistic insight into the beneficial cardiovascular effects of HO-1 and CO and also point to a novel signalling pathway which can be targeted in future treatment strategies for vascular diseases.
